# The Q Motif Is Involved in DNA Binding but Not ATP Binding in ChlR1 Helicase

**DOI:** 10.1371/journal.pone.0140755

**Published:** 2015-10-16

**Authors:** Hao Ding, Manhong Guo, Venkatasubramanian Vidhyasagar, Tanu Talwar, Yuliang Wu

**Affiliations:** Department of Biochemistry, University of Saskatchewan, Health Sciences Building, 107 Wiggins Road, Saskatoon, Saskatchewan, Canada; University of Iowa, UNITED STATES

## Abstract

Helicases are molecular motors that couple the energy of ATP hydrolysis to the unwinding of structured DNA or RNA and chromatin remodeling. The conversion of energy derived from ATP hydrolysis into unwinding and remodeling is coordinated by seven sequence motifs (I, Ia, II, III, IV, V, and VI). The Q motif, consisting of nine amino acids (GFXXPXPIQ) with an invariant glutamine (Q) residue, has been identified in some, but not all helicases. Compared to the seven well-recognized conserved helicase motifs, the role of the Q motif is less acknowledged. Mutations in the human *ChlR1* (*DDX11*) gene are associated with a unique genetic disorder known as Warsaw Breakage Syndrome, which is characterized by cellular defects in genome maintenance. To examine the roles of the Q motif in ChlR1 helicase, we performed site directed mutagenesis of glutamine to alanine at residue 23 in the Q motif of ChlR1. ChlR1 recombinant protein was overexpressed and purified from HEK293T cells. ChlR1-Q23A mutant abolished the helicase activity of ChlR1 and displayed reduced DNA binding ability. The mutant showed impaired ATPase activity but normal ATP binding. A thermal shift assay revealed that ChlR1-Q23A has a melting point value similar to ChlR1-WT. Partial proteolysis mapping demonstrated that ChlR1-WT and Q23A have a similar globular structure, although some subtle conformational differences in these two proteins are evident. Finally, we found ChlR1 exists and functions as a monomer in solution, which is different from FANCJ, in which the Q motif is involved in protein dimerization. Taken together, our results suggest that the Q motif is involved in DNA binding but not ATP binding in ChlR1 helicase.

## Introduction

Helicases are classically defined as motor proteins that move directionally along a nucleic acid phosphodiester backbone, separating two annealed nucleic acid strands (i.e., DNA, RNA, or DNA-RNA hybrid) using energy derived from ATP hydrolysis. They move directionally along a nucleic acid strand of the duplex, which identifies them as either 5'→3' helicases or 3'→5' helicases. Helicases play roles in virtually all aspects of nucleic acid metabolism, including DNA replication, repair, recombination, transcription, chromosome segregation, and telomere maintenance [1–3]. According to their substrates, helicases are also classified as DNA or RNA helicases. Because of their biological importance, helicase defects have been linked to various genetic disorders and cancers [4–6].

Based on the presence and the form of helicase motifs, helicases have been classified into six superfamilies [7], of which superfamily 2 (SF2) is the largest [2]. The SF2 helicases have seven signature motifs (I, Ia, II, III, IV, V, and VI) that coordinate the conversion of energy derived from nucleoside triphosphate (NTP) hydrolysis into unwinding of double-stranded nucleic acids [7]. These motifs are usually clustered in a region of 200 to 700 amino acids called the helicase core domain. Because of the sequence of motif II, helicases are also divided into DEAD, DEAH, or DEXH-box protein families [8]. Structural and biochemical evidence suggests that motifs I, II, and VI are involved in ATP binding and hydrolysis; motifs Ia, IV, and V are important for DNA binding; and motif III is responsible for coupling ATP hydrolysis to unwinding and remodeling events. X-ray crystallographic studies demonstrate that the conserved helicase motifs are located in close proximity, suggesting that they form a large functional domain coordinating ATP binding and hydrolysis for DNA or RNA unwinding [7,8].

The Q motif (Gly-Phe-X-X-Pro-X-Pro-Ile-Gln) was first identified in DEAD box RNA helicases a decade ago [9]. It is located 17 amino acids upstream of motif I and consists of a nine amino acid sequence with an invariant glutamine (Gln, Q) residue. Since its discovery, the presence and functionality of the Q motif have been reported in SF1 helicases, UvrD [10,11], PcrA [12], and Rep [13]; DExH-box protein, UvrB [14], DHX9, and DDX20 [15]; DEAD-box RNA helicases, Dhh1 [16], Dbp5/DDX19 [17], and Hera [18,19]; and many other helicase domain-containing proteins, e.g., reverse gyrase [20], phage λ packaging motor [21], and endonuclease EcoP15I [22]. Therefore, the Q motif is present not only in DEAD-box proteins, but also helicase domain-containing ATPases.

Site-specific mutagenesis studies demonstrate that the Q motif controls ATP binding and hydrolysis in the yeast translation-initiation factor / RNA helicase eIF4A [9], a viral helicase NS3 [23], and *Mycobacterium tuberculosis* RecG helicase [24]. The Q motif in yeast RNA helicase Ded1 not only regulates ATP binding and hydrolysis but also exhibits an affinity for RNA substrates [25]. It was further proposed that the Q motif in eIF4A and Ded1 RNA helicases functions as a molecular on-off switch for ATP hydrolysis and helicase activity [26]. In a study of the RNA helicase Hera, the Q motif was found to be responsible for sensing the nucleotide state of the helicase and establishing a stable interaction between motif I and other helicase motifs, the latter being a requirement for catalytic competence [19]. However, a study of helicase domain-containing SNF2 (sucrose non-fermentable 2) protein SMARCAL1 suggests that the Q motif is required for ATP hydrolysis but not ATP binding [27]. Thus, the Q motif has been found in helicases beyond the DEAD-box family; however, the role of the Q motif in helicases is inconclusive.

ChlR1 (also known as DDX11) is a DEAH-box 5' →3' DNA helicase first discovered in budding yeast, and its functions appear to be conserved throughout evolution [28]. RNAi-dependent knock down of ChlR1 causes premature sister chromatid separation and a profound delay in mitotic progression in human cells, suggesting that ChlR1 is required to establish proper sister chromatid cohesion during S phase [29]. ChlR1-null mice die on embryonic day 10.5 due to the loss of sister chromatid cohesion [30]. Mutations in human ChlR1 are genetically linked to Warsaw Breakage syndrome (WABS), which is characterized by severe microcephaly, pre- and post-natal growth retardation, and abnormal skin pigmentation [31]. In a patient diagnosed with WABS who has compound heterozygous mutations in ChlR1, a splice site mutation and a 3-bp in-frame C-terminal deletion (c.2689_2691del [p.K897del]) were shown to abrogate the ChlR1 helicase activity [32]. Since then, a further three siblings with consanguineous parents were identified as having a novel homozygous mutation in ChlR1 [33]. Recently, a third case was reported that shares similar phenotypic features to the previously reported cases [34].

ChlR1 has been shown to interact with forked duplex DNA and efficient unwinds the 5’ flap structure, a key intermediate of lagging strand processing [32]. Recently, we found that ChlR1 preferably unwinds triplex DNA [35]. ChlR1 can also interact with Fen1 and stimulate its 5’ flap endonuclease activity [36]. A recent study reports that the ChlR1 WABS missense mutation (R263Q) located in the conserved Fe-S domain impaired helicase activity by perturbing its DNA binding and DNA-dependent ATP hydrolysis [33]. Chl1 (yeast ChlR1 homolog) promotes Scc2 (component of cohesion complex) loading onto DNA while Chl1 mutant cells fail to recruit Scc2 with DNA, resulting in chromatid cohesion enrichment [37]. Both Chl1 expression and chromatin-recruitment are tightly regulated through the cell cycle, peaking during S-phase. Sequence alignment of ChlR1 with its homologs revealed that ChlR1 helicase contains the Q motif (**S1 Fig**); however, the role of the Q motif in ChlR1 remains unknown.

## Materials and Methods

### Plasmid DNA

Human *ChlR1* cDNA was cloned into the *Hind*III and *Xho*I sites of pcDNA3 with 3× FLAG tag at the C-terminus [35]. The Q23A mutation was generated by a QuikChange site-directed mutagenesis kit (Stratagene) according to the manufacturer's instructions using the designed mutagenic primers shown in **S1 Table**. All plasmids were sequenced to verify that no undesired mutations were introduced during PCR and cloning.

### DNA substrates

PAGE-purified oligonucleotides used for the preparation of DNA substrates, DC26, and T_STEM_25 were purchased from IDT (Integrated DNA Technologies, **S1 Table**). The duplex, triplex, and G-quadruplex substrates (OX-1 G2’) were 5′-^32^P-end-labeled with T4 polynucleotide kinase (NEB), and prepared as described previously [35];[38].

### Recombinant protein

The PEI transfection method [39] was used for transfecting ChlR1 plasmid DNA into HEK293T cells, and the recombinant ChlR1 proteins were purified as per a previously described protocol [35] with modifications. Briefly, cell pellets were resuspended in buffer A [10 mM Tris HCl (pH 7.4), 10 mM KCl, 1.5 mM MgCl_2_, 1 mM DTT, 0.5 mM PMSF, proteinase inhibitors]. Cells were lysed in the presence of protease inhibitors (Roche) for 30 min at 4°C with mild agitation and centrifuged at 43,500 g for 30 min at 4°C. The supernatant was incubated with FLAG antibody resin (Sigma) for 2 h at 4°C. The resin was washed twice with buffer B [20 mM Tris HCl (pH 7.4), 500 mM NaCl, 10% glycerol, 0.5% Nonidet P-40, 1.5 mM MgCl_2_, 0.2 mM EDTA]. ChlR1 was eluted with 4 μg/mL of 3 × FLAG peptide (Sigma) in buffer C [25 mM Tris HCl (pH 7.4), 100 mM NaCl, 10% glycerol, 0.1% Tween 20, 5 mM Tris (2-carboxyethyl) phosphine hydrochloride] for 1 h. Aliquots were frozen in liquid nitrogen and stored at -80°C. The concentrations of wild-type (WT) and mutant ChlR1 proteins were determined by Bradford assay (Bio-Rad) using bovine serum albumin (BSA) as a standard.

### Size exclusion chromatography (SEC)

Purified recombinant ChlR1 protein was applied to a size exclusion column (Sephacryl S-300 HR, GE Healthcare) using an AKTApure protein chromatography system (GE Healthcare) equilibrated and eluted with 25 mM Tris·HCl (pH 7.5), 10% glycerol, 0.15 M NaCl, 1 mM EDTA, and 0.5 mM DTT. The column was run at a flow rate of 0.1 mL/min, and fractions of 0.5-mL were collected. The protein fractions were detected using a UV detector. The size exclusion column was calibrated using standard molecular mass markers (Sigma), namely, Blue Dextran (2000 kDa), thyroglobulin (669 kDa), apoferritin (443 kDa), beta amylase (200 kDa), alcohol dehydrogenase (150 kDa), and albumin (66 kDa).

### Western blot

Protein was denatured at 100°C for 5 min, then resolved on 10% polyacrylamide gels in Tris-glycine SDS buffer and transferred to PVDF membranes. The membrane was blocked in PBS containing 5% powdered milk at room temperature for 1h, followed by probing with rabbit polyclonal anti-ChlR1 (1:2000, Abnova) or anti-FLAG antibody (1:5000, Sigma), respectively. Goat anti-rabbit or goat anti-mouse IgG-horseradish peroxidase conjugate (Santa Cruz Biotech) was used as a secondary antibody at a 1:10,000 dilution and detected using ECL Plus (GE Healthcare).

### Helicase assays

Helicase assay reaction mixtures (20 μL) contained 40 mM Tris HCl (pH 7.4), 25 mM KCl, 5 mM MgCl_2_, 2 mM dithiothreitol, 2% glycerol, 100 ng/uL BSA, 2 mM ATP, 0.5 nM of DNA substrate (forked duplex or G4 substrate), and the indicated concentrations of ChlR1. Helicase reactions were initiated by the addition of ChlR1, incubated at 37°C for 20 min, and terminated by addition of Stop buffer containing EDTA. Reaction products were resolved on nondenaturing 12% (19:1 acrylamide/bisacrylamide) polyacrylamide gels for the forked duplex substrates and nondenaturing 8% (19:1 acrylamide/bisacrylamide) polyacrylamide gels, as described previously [32]. For triplex DNA, helicase assay reaction mixtures (20 μL) contained 25 mM HEPES (pH 7.5), 25 mM potassium acetate, 1 mM magnesium acetate, 1 mM DTT, 100 μg/mL BSA, 1 mM ATP, 0.5 nM of the specified triplex DNA, and the indicated concentrations of ChlR1 protein. The products of the helicase reactions were resolved on nondenaturing 10% (19:1 acrylamide:bisacrylamide) polyacrylamide gels with 40 mM Tris acetate (pH 5.5) and 25% glycerol and running buffer containing 40 mM Tris acetate, pH 5.5, and 5 mM MgCl_2_. Radiolabeled DNA species in polyacrylamide gels were visualized using a PharosFX Imager and quantified using Quantity One software (Bio-Rad).

### Electrophoretic mobility shift assay (EMSA)

Protein/DNA binding mixtures (20 μL) contained the indicated concentrations of ChlR1 and 0.5 nM of the specified ^32^P-end-labeled DNA substrate in the same reaction buffer as used for helicase assays (see above) without ATP. The binding mixtures were incubated at room temperature for 30 min after the addition of ChlR1. After incubation, 3 μL of Loading dye (74% glycerol, 0.01% xylene cyanol, 0.01% bromphenol blue) were added to each mixture, and samples were loaded onto native 5% (19:1 acrylamide/bisacrylamide) polyacrylamide gels and electrophoresed at 200 V for 2 h at 4°C using 1×TBE as the Running buffer. The resolved radiolabeled species were visualized using a Phosphor-Imager and analyzed with Quantity One software.

### ATP hydrolysis assays

ATP hydrolysis was measured using [γ-^32^P] ATP (PerkinElmer) and analysis by thin-layer chromatography (TLC) on polyethyleneimine-cellulose plates (J.T. Baker). The standard reaction mixture (20 μL total volume) contained 25 mM Hepes-NaOH (pH 7.5), 25 mM potassium acetate, 1 mM magnesium acetate, 1 mM DTT, 100 μg/mL BSA, 250 μM [γ-^32^P] ATP, and 60 nM ChlR1 protein and was incubated at 37°C for 0–45 min. Reactions were quenched with EDTA to a final concentration of 50 mM. The reaction mixture was spotted onto a PEI-cellulose TLC plate and resolved using 0.5 M LiCl and 1 M formic acid as the carrier solvent. The TLC plate was exposed to a phosphorimager cassette for 1 h and visualized using a Phosphor-Imager and analyzed with Quantity One software.

For experiments to determine the Michaelis constant Km (ATP), M13mp18 ssDNA concentration was 2.1 nM, the concentration of ATP ranged from 31 to 4000 μM, and the reaction was incubated for 30 min. For determination of k_cat_, the concentration of ATP was 8.5 mM. Aliquots of 5μL were removed and quenched with 5 μL of 0.1 M EDTA at 0, 7.5, 15, 30, and 45 min. The kinetic parameters were calculated by Enzyme Kinetics 1.3 (SigmaPlot 11.2, Systat Software Inc) using the Michaelis-Menton equation. All experiments were repeated at least three times and data presented are the mean with standard deviation (SD, error bars).

### ATP binding assays

Two methods were used to determine of ChlR1 protein ATP binding activity. An ATP AffiPur kit (cat# AK-102, Jena Bioscience) was used to detect ATP binding according to the manufacturer’s protocol. Equal amounts of purified protein (100 μg) were incubated with 50 μL of ATP-agarose beads rotated at 4°C for 2 h. The resulting beads were washed 3 times with 500 μL wash buffer, and bound protein was eluted by adding SDS-PAGE sample buffer and heated at 100°C for 5 min. After centrifuging at 16,873 g for 10 min, the supernatant was subjected to Western blot analysis using anti-FLAG and anti-ChlR1 antibodies.

For the ATP quantitative binding assay, the reaction (30 μL) was performed in the same reaction buffer as described above for the helicase or ATPase assays with 5 μCi [α-^32^P] ATP (3000 Ci/mmol, PerkinElmer). Assays were initiated by adding ChlR1 protein to a final concentration of 230 nM, followed by incubating at 4°C for 30 min. Reactions were then applied to Bio-Spin P30 Tris chromatography columns (Bio-Rad) that had been pre-equilibrated in a reaction buffer. One drop (~45 μL) fractions were collected as flow-through under gravity from columns eluted with TE buffer. The specific radio activity of each fraction was determined by a liquid scintillation counter (Beckman LS 6000TA). The first peak (3–4 drops) was considered as protein-bound ATP, and the second peak as unbound ATP.

### Steady state rotational anisotropy assays

Steady state fluorescence depolarization (rotational anisotropy) was used to measure enzyme-DNA binding affinities using the same fluorescence labeled forked double stranded (ds) DNA substrates used for the helicase assays (**S1 Table**). Measurements were performed in a quartz cuvette of 1 cm path length. Reactions of 70 μL contained F-labeled forked dsDNA (10 nM) in helicase assays buffer (without ATP); ChlR1-WT (0–1900 nM) or ChlR1-Q23A (0–2300 nM) was added via titration. A QuantaMaster QM-4 spectrofluorometer (Photon Technology International) with a dual emission channel was used to collect data and calculate anisotropy. Measurements were made at 21°C. Samples were excited with vertically polarized light at 495 nm (6 nm band pass) and vertical and horizontal emissions were measured at 520 nm (6 nm band pass). Apparent dissociation constants (*Kd*) were obtained by fitting to a sigmoidal curve using Sigma Plot 11.2 software.

### Thermal stability shift assay (TSA)

All reactions were incubated in a 30 μL final volume and assayed in 96-well plates using 20 × SYPRO Orange (Invitrogen) and 1 μM purified ChlR1 protein. Protein elution buffer was added instead of protein in the control samples. The plates were sealed with Optical-Quality Sealing Tape (Bio-Rad). Thermal melting experiments were carried out using a StepOnePlus Real-Time PCR System (Applied Biosystems) melt curve program with a ramp rate of 1°C and temperature range of 25 to 60°C. Melting temperature (Tm) was defined as the midpoint temperature of the protein-unfolding transition, and calculated by fitting the sigmoidal melt curve to the Boltzmann equation (with R^2^ values of >0.99).

### Proteolysis mapping

Equal amounts of ChlR1-WT or ChlR1-Q23A protein (300 nM) were used in digestion reactions (20 μL), then incubated at room temperature for 3 min with a range of trypsin (0–200 nM, Sigma). Reactions were stopped by adding 10 μL of SDS-PAGE gel loading buffer, and electrophoresed on a 10% SDS-PAGE followed by Western blotting using an anti-FLAG antibody (Sigma).

### Filter-binding assay

Filter-binding experiments were performed in a 96-well dot blot apparatus (Bio-Rad). For ATP binding, 10 nM AMP-PNP and 0.84 nM [α-^32^P] ATP were used; for DNA binding, 10 nM non-labelled fork duplex and 0.17 nM radiolabeled fork duplex were used. The reaction was incubated with the indicated concentration of ChlR1 proteins (WT and Q23A) in a 50 μL total volume of binding buffer (25 mM Tris-HCl, pH 7.5, 25 mM KOAC, 1 mM Mg(OAC)_2_, 1 mM DTT, 100 μg/mL bovine serum albumin) at 37°C for 30 min. The mixtures were then filtered through a membrane sandwich containing a top layer of protein nitrocellulose membrane (Millipore) and three bottom layers of filter paper (Whatman). Before sample application, the filter was pretreated with 200 μL of the binding buffer. After application of the samples, the filters were washed twice with 100 μL of washing buffer (25 mM Tris-HCl, pH 7.5, 25 mM KOAC, 1 mM Mg(OAC)_2_), the membranes were separated and dried at room temperature for 5 min, and quantified using a PhosphorImager (Bio-Rad). The data were analyzed and the *Kd* value was determined using GraphPad Prism software.

### Sucrose density gradient centrifugation

A linear sucrose gradient (10–25%, wt/vol) was prepared in a buffer containing 25 mM Tris (pH7.4), 150 mM NaCl, 1 mM DTT using 11×60-mm centrifugation tubes (Beckman). The gradients were stored for at least 1 h at 4°C before they were loaded with ChlR1 proteins (0.5 mg/mL; 50 μL) and centrifuged at 120,000 g for 16 h in an SW Ti60 rotor (Beckman) at 4°C. After centrifugation, fractions of 100 μL were collected from the top and analyzed by 10% SDS-PAGE. Standard globular proteins (alcohol dehydrogenase, 150 kDa; BSA, 66 kDa; and carbonic anhydrase, 29 kDa) were run in parallel. The molecular mass of ChlR1 proteins were calculated using the formula M = fSNa/(1-νρ), where f is Stokes radius of protein, S is Svedberg unit of the protein, Na is Avogadro number, ν is specific volume of the protein and ρ is the density of solution.

## Results

### ChlR1-Q23A protein abolishes helicase activity

**Fig 1:**
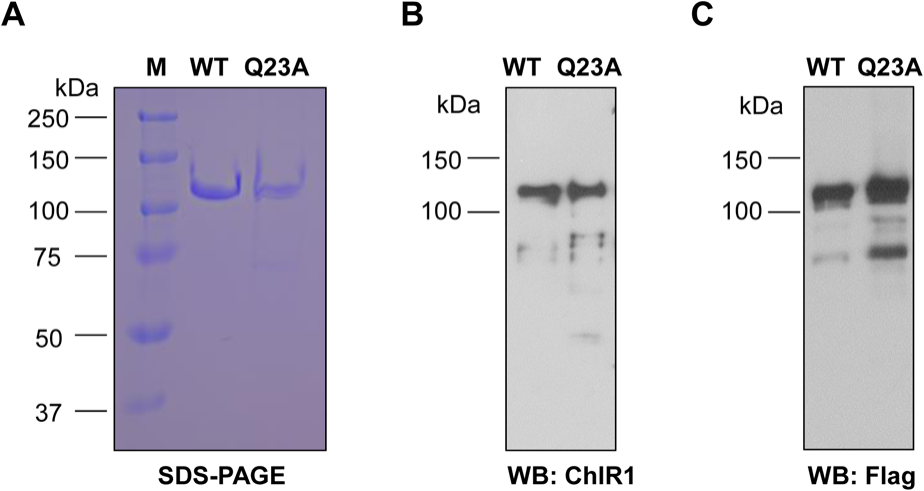
Purification and identification of ChlR1 proteins. (**A**) ChlR1 proteins (WT and Q23A) were purified and electrophoresed on SDS-polyacrylamide gel and stained with Coomassie blue. (**B-C**) Western-blot analysis of the purified proteins with antibodies ChlR1 (**B**) and FLAG (**C**).

Close inspection of human ChlR1 and its orthologs across species revealed a conserved glutamine (Q motif) residue upstream of motif I (**S1 Fig**). To determine the functions of the Q motif in ChlR1 protein, we changed the glutamine to alanine (designated ChlR1-Q23A) and transfected the plasmid to HEK 293T cells using PEI. The wild-type and mutant ChlR1 proteins were purified to near homogeneity as judged by their appearance as single bands after electrophoresis on Coomassie blue-stained SDS-polyacrylamide gel (**Fig 1A**). The protein identity was confirmed by Western blot using an anti-ChlR1 antibody and anti-FLAG antibody, respectively (**Fig 1B and 1C**).

**Fig 2:**
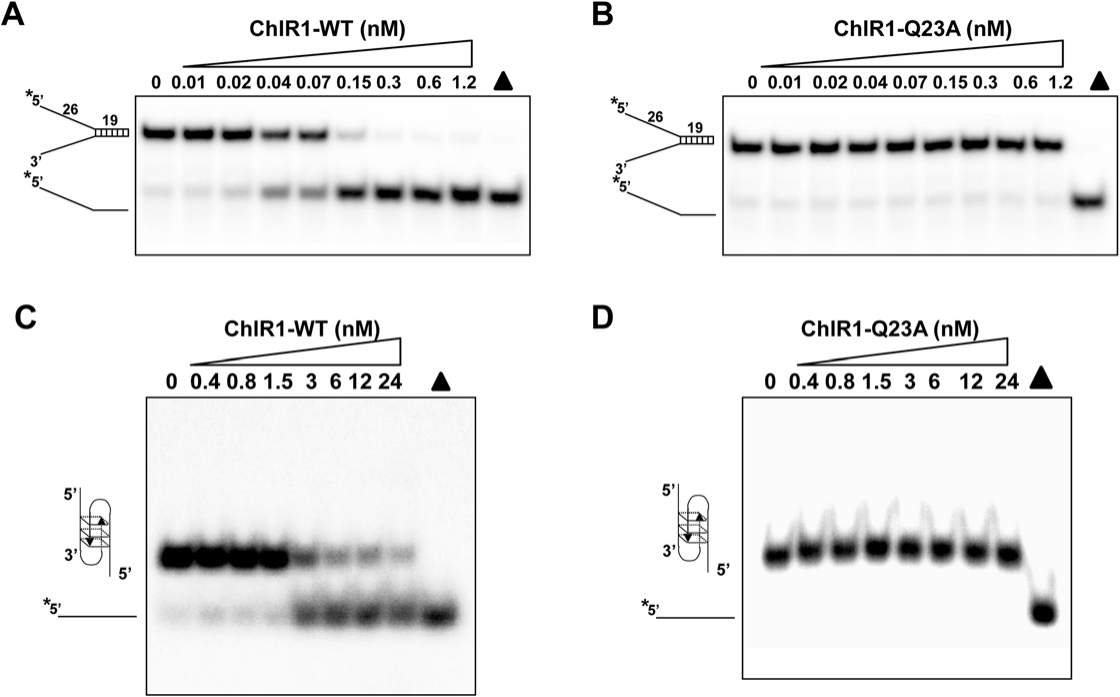
Helicase analysis of ChlR1 proteins on forked duplex DNA and G4 DNA. Helicase reactions were performed by incubating with indicated protein concentration and 0.5 nM duplex DNA substrate (**A-B**) or OX-1 G2’ DNA substrate (**C-D**) at 37°C for 20 min. The triangle indicates heat denatured DNA substrate control.

Using a forked duplex DNA substrate that we know ChlR1-WT can efficiently unwind [32,35], we examined the helicase activity of ChlR1-Q23A. The results revealed that changing the invariant glutamine to alanine of the Q motif abolished ChlR1’s unwinding activity on forked duplex DNA (**Fig 2A and 2B**). Approximately 100% of the forked duplex was unwound at the highest concentration of 1.2 nM ChlR1-WT; however, ChlR1-Q23A failed to unwind the duplex substrate at the same concentration. We recently found that ChlR1 helicase has a strong preference for a two-stranded antiparallel G4 substrate (G2’) [32]; thus, we also examined the ChlR1-Q23A unwinding activity of this substrate. ChlR1-WT was able to unwind more than 60% of the substrate at its highest concentration (24 nM); however, ChlR1-Q23A failed to unwind this substrate at the same concentration (**Fig 2C and 2D**). We also increased the incubation time (60 min), ATP concentration (10 mM), or protein concentration (to 12 nM for forked duplex DNA and 240 nM for G2’ substrate) but failed to detect any unwinding activity for ChlR1-Q23A (data not shown). Taken together, we concluded that the ChlR1-Q23A mutant abolishes the helicase activity.

### ChlR1-Q23A protein has poor DNA binding ability

**Fig 3:**
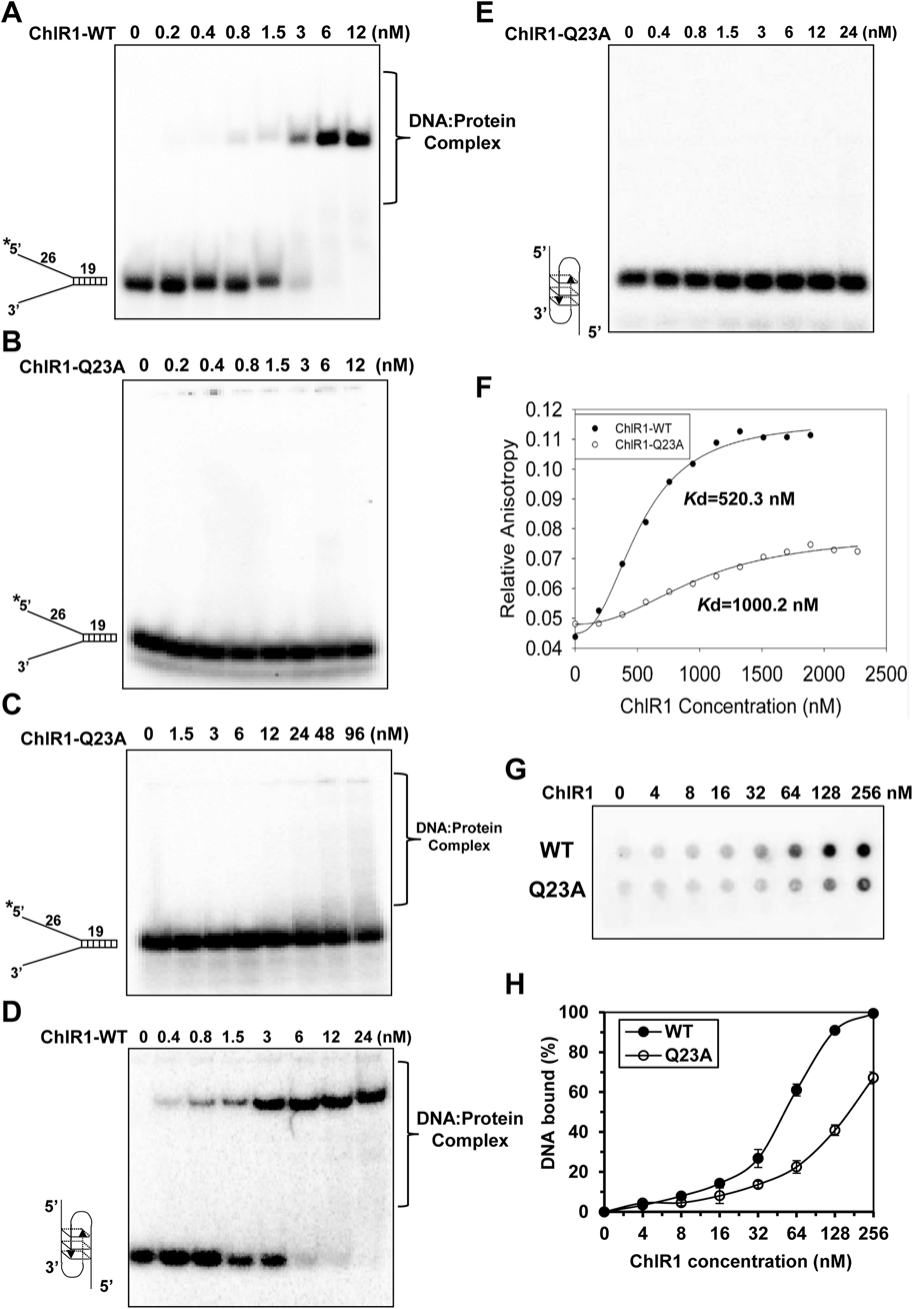
DNA binding analysis of ChlR1 proteins on forked duplex DNA and G4 DNA. The indicated concentrations of ChlR1 proteins were incubated with 0.5 nM forked duplex DNA substrate (**A-C**) or OX-1 G2’ DNA substrate (**D-E**) at room temperature for 30 min under standard EMSA conditions as described in “Materials and methods”. The DNA-protein complexes were resolved on native 5% polyacrylamide gels. (**F**) Variation of fluorescence anisotropy as a function of ChlR1 protein concentration. (**G**) A representative image of filter dot blot assays of ChlR1 proteins binding forked duplex DNA. (**H**) Quantitative analyses of DNA bound to ChlR1 proteins in panel G. Data represent the mean of at least three independent experiments with SD indicated by error bars.

The ability of the ChlR1-Q23A mutant protein to abolish helicase activity might reflect impaired DNA binding activity. To test this assumption, we performed electrophoretic mobility shift assays with ChlR1-WT and ChlR1-Q23A using radiolabeled DNA substrates that were used in the helicase assay. Results demonstrated that ChlR1-WT bound the DNA molecules in a protein concentration-dependent manner while the binding ability of the ChlR1-Q23A protein was abolished (**Fig 3A and 3B**). When the protein concentration was increased by up to eight times (96 nM), ChlR1-Q23A could slightly bind the substrate (**Fig 3C**). Compared with forked duplex DNA, ChlR1-WT had a lower binding ability to the G2’ substrate; however, ChlR1-Q23A completely lost its binding ability to this G2’ structure (**Fig 3D and 3E**), even at an increased protein concentration (240 nM, data not shown). Thus, we concluded that the Q motif is essential for ChlR1 protein’s DNA binding ability.

To examine the binding ability of ChlR1 proteins with DNA by an alternative method, we performed rotational anisotropy assays to determine the *Kd* value. Using the same forked duplex substrate used in our EMSA, we labelled DNA with fluorescein (**S1 Table**), and incubated a consistent amount of DNA with an increasing concentration of protein (**Fig 3F**). The apparent *Kd* for ChlR1-Q23A was 1000.2 nM, which is two-fold higher than the *Kd* of ChlR1-WT (520.3 nM). Additionally, we performed filter binding assays of ChlR1 proteins with DNA (**Fig 3G**) and again determined the *Kd* values for WT of 105.5 nM, and for Q23A of 397.9 nM (**Fig 3H**), further implicating that Q23A mutant has a reduced affinity for DNA.

Binding of ATP might cause helicase conformational changes [40–42], which in turn affects their DNA binding. For example, the ATP bound form of ADAAD protein has a higher affinity for ssDNA [27]. Thus, we pre-incubated ChlR1 protein with AMP-PNP, a non-hydrolytic ATP analogy, and then incubated with forked dsDNA. We found that ATP binding did not stimulate ChlR1’s DNA binding ability (**S2 Fig**).

### ChlR1-Q23A is impaired for ATP hydrolysis but retains ATP binding

**Fig 4:**
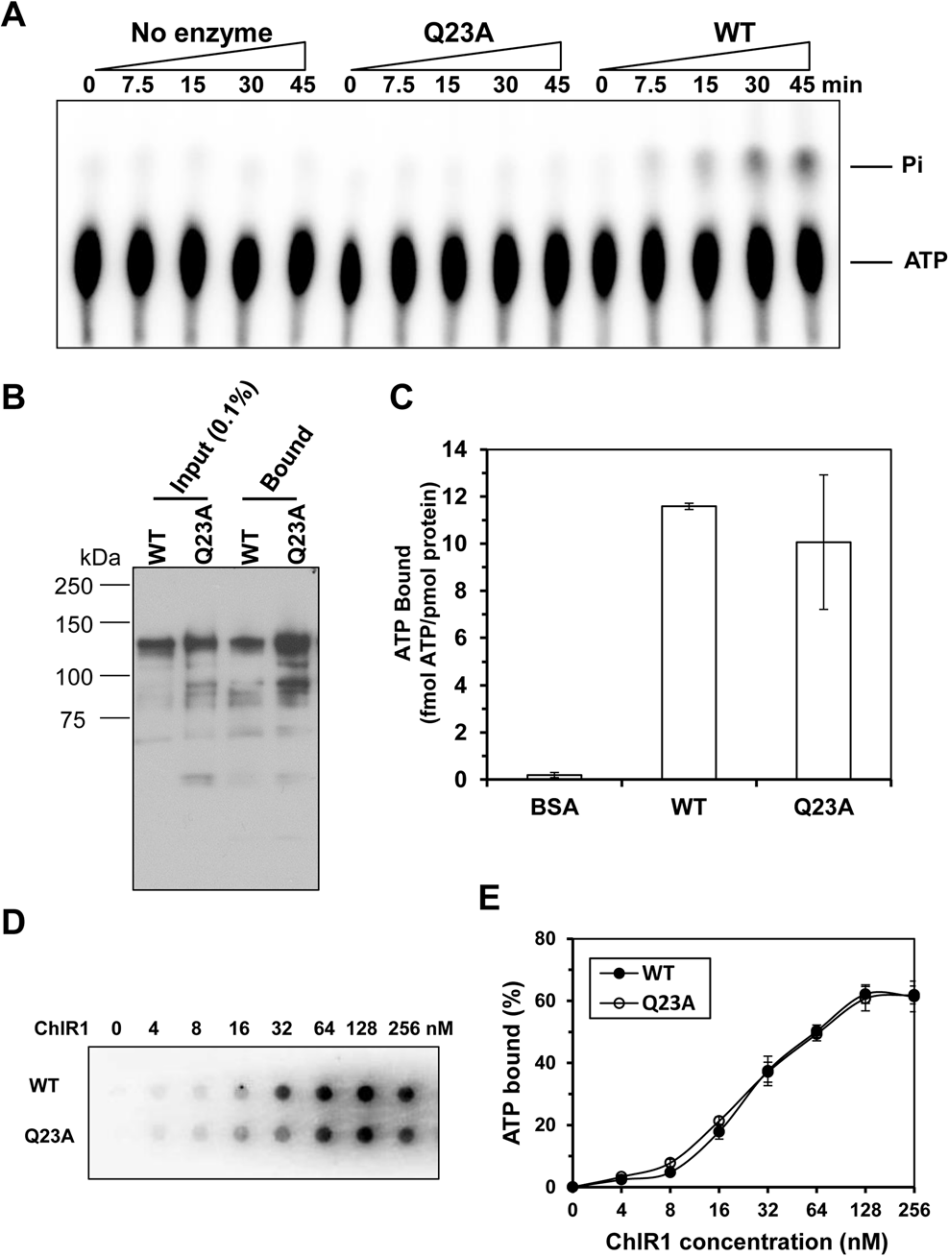
ATP hydrolysis and ATP binding assays of ChlR1 proteins. (**A**) A representative image of ChlR1 ATP hydrolysis detected by TLC. (**B**) ATP binding by ChlR1 proteins was determined by ATP agarose (Jena Bioscience) as described in “Materials and methods”, followed by Western blot with an anti-FLAG antibody. (**C**) ATP binding by wild-type ChlR1 and mutant protein. α^32^P-ATP binding to ChlR1-WT and ChlR1-Q23A was performed by gel filtration chromatography as described in “Materials and methods”. The same amount of protein was used, and the total amount of bound ATP was divided by protein and presented as fmol ATP per pmol protein. BSA was used as a control. (**D**) A representative image of filter dot blot assays of ChlR1 proteins binding α^32^P-ATP. (**E**) Quantitative analyses of ATP bound to ChlR1 proteins in panel D. Data represent the mean of at least three independent experiments with SD indicated by error bars.

Most helicases have DNA- or RNA-dependent ATP hydrolysis activity. Thus, we examined the DNA-dependent ATPase activity of ChlR1-Q23A and compared it with ChlR1-WT. Using covalently closed M13 single strand DNA as the effector molecule and ATP ranging from 31 to 4000 μM, we determined the *Km* value of ATP hydrolysis for ChlR1-WT is 415.7 ± 58.4 μM. Because of the very low ATPase activity, the *Km* for ChlR1-Q23A could not be determined (**Fig 4A**). Using an ATP concentration (8.5 mM) that was ∼20-fold greater than the *Km* for ChlR1-WT, we performed ATPase assays for ChlR1-WT and ChlR1-Q23A and determined the *k*_cat_ values for these two proteins were 1073.3 ± 46.7 and 110.7 ± 9.3 s^-1^, respectively. These results suggest that the ChlR1-Q23A mutation seriously compromises the ability of ChlR1 to hydrolyze ATP.

Next, we asked whether ChlR1-Q23A was able to bind ATP. We used an ATP AffiPur kit, in which we incubated the same amount of ChlR1-WT or -Q23A proteins with ATP-agarose. It revealed that similar levels of WT and Q23A proteins were retained in ATP beads as detected by Western blot (**Fig 4B**). Furthermore, to quantitatively compare ATP binding, an equal amount of Q23A or WT proteins was incubated with [α-^32^P] ATP under identical conditions, and binding mixtures were analyzed by gel filtration chromatography. Scintillation counting of the eluted fractions demonstrated that ChlR1-Q23A bound ATP similarly to ChlR1-WT. 1 pmol of ChlR1-WT protein bound 11.6 ± 0.13 fmol ATP, while 1 pmol ChlR1-Q23A bound 10.1 ± 2.86 fmol ATP (**Fig 4C)**. Additionally, we performed filter binding assays of ChlR1 proteins with [α-^32^P] ATP (**Fig 4D**), and determined the *Kd* of WT is 51.78 nM, and of Q23A is 43.8 nM (**Fig 4E**). From these results, we concluded that ChlR1-Q23A could bind ATP similarly to ChlR1-WT but did not efficiently hydrolyze the nucleotide in the presence of a DNA effector.

### ChlR1-Q23A has no translocase activity

**Fig 5:**
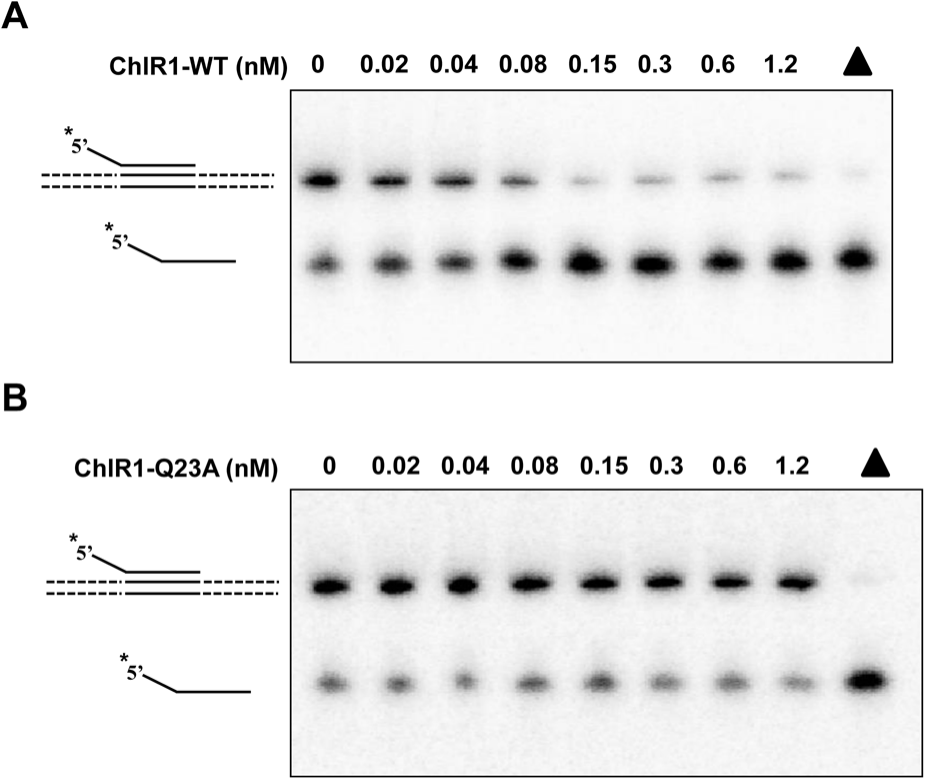
ChlR1-Q23A fails to unwind DNA triple helixes. **(A-B)** Helicase reactions (20 μL) were performed by incubating the indicated ChlR1-WT (**A**) or ChlR1-Q23A (**B**) concentrations with 0.5 nM 5’ tail plasmid-triplex substrate at 37°C for 20 min under standard helicase assay conditions as described in “Materials and methods”. Triangle indicates heat-denatured DNA substrate control.

Triplex displacement experiments have been utilized to monitor the translocase activity of helicases, including AddAB [43] and FANCM [44]. In this assay, a triple helix is formed when a third strand forms Hoogsteen base pairs with duplex DNA. If a translocase proceeds through the triplex, it will displace the third strand. We found that wild-type ChlR1, but not its Q mutant Q23A, displayed triple-helix displacement activity (**Fig 5A and 5B**). Under the same reaction conditions, ChlR1-WT was also able to unwind a short triplex structure (named flush triplex), which was constructed by annealing the same pyrimidine motif third strand (TC30) to a 30 bp duplex fragment; however, the mutant ChlR1-Q23A failed in this regard (**S3 Fig**). Together, these results suggest that ChlR1 can dissociate DNA triplexes; however, the Q23A mutant has no translocase activity.

### ChlR1-Q23A has a similar globular structure as ChlR1-WT, but some subtle conformational differences

**Fig 6:**
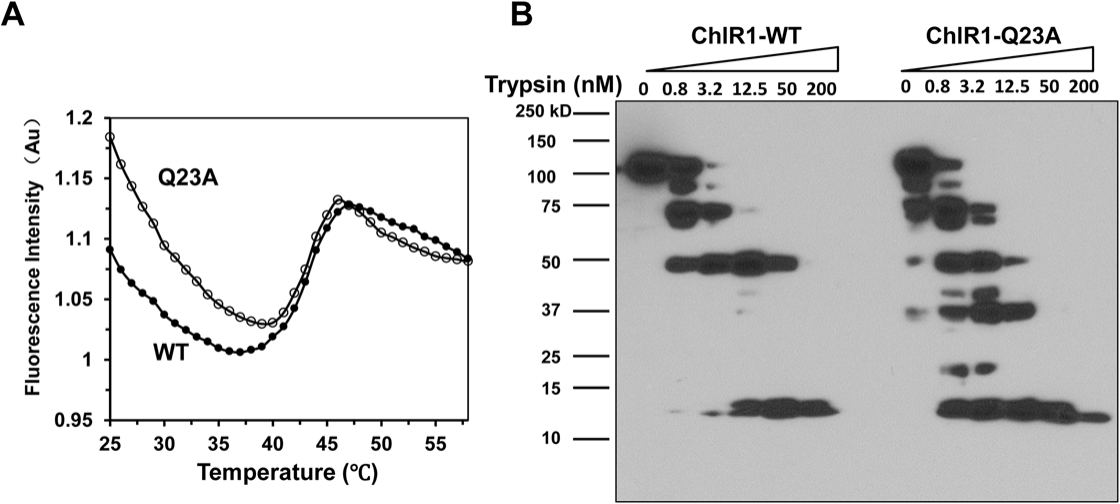
Thermal stability assays and partial proteolysis mapping of ChlR1 proteins. (**A**) Unfolding curves of ChlR1-WT and ChlR1-Q23A over a temperature range from 25 to 60°C. (**B**) Representative image of partial proteolysis mapping of ChlR1 proteins. Purified ChlR1 proteins (WT and Q23A) were digested with increasing trypsin concentration, and protein fragments were separated on SDS-PAGE followed by Western blot analysis using an anti-FLAG antibody.

To address whether ChlR1-Q23A protein has a similar structure to the wild type, we attempted to use circular dichroism to determine the secondary structure and folding properties of proteins but failed because circular dichroism analysis requires a significant amount of concentrated protein and specific buffer. In contrast, thermal shift assays are widely used to measure the thermal stability of target proteins [45–47], which indirectly reflect protein folding. Thus, we performed a thermal shift assay for the purified recombinant ChlR1 proteins. In the temperature range from 25 to 60°C, we found that ChlR1-Q23A protein exhibited a similar transition curve to the wild-type protein (**Fig 6A**). Melting curve analysis with a derivative reporter showed that the midpoint (*Tm*) for both ChlR1-WT and ChlR1-Q23A was 43.9°C, with a Δ*T*_*m*_ of ~0°C, suggesting that ChlR1-Q23A protein’s globular structure is similar to that of ChlR1-WT.

In order to gain insight into the subtle conformational change that may be caused by the Q23A mutant, we used partial proteolysis mapping to probe the physical architecture of ChlR1 proteins. With increasing trypsin concentration, both ChlR1-WT and -Q23A yielded some stable fragments of around 75, 50, and between 10–15 kDa, but ChlR1-Q23A yielded additional fragments around 37 and 15 kDa (**Fig 6B**). These results demonstrate that, even though ChlR1-WT and ChlR1-Q23A have similar globular structures; some subtle conformational differences between these two proteins exist and may be caused by Q23 residue substitution.

### ChlR1 exists and functions as a monomer

**Fig 7:**
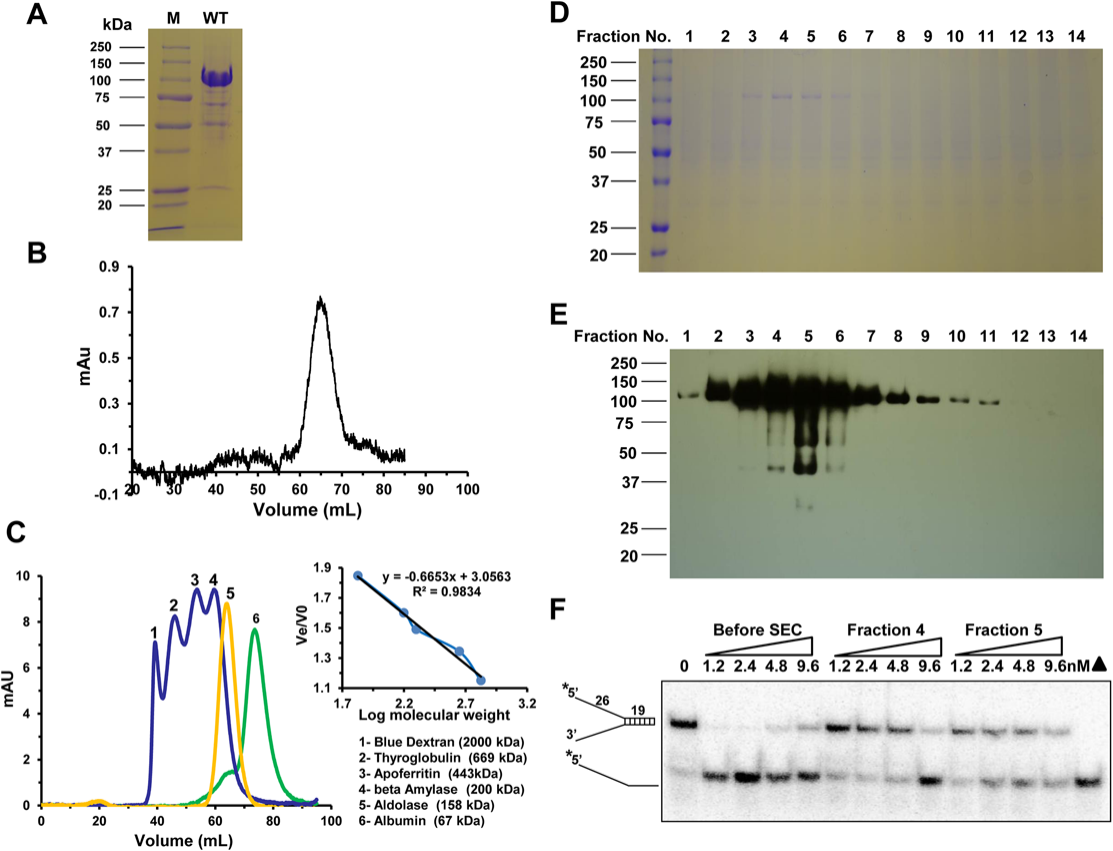
Determination of ChlR1 protein oligomerization state. (**A**) Coomassie blue stained SDS-PAGE gel showing the ChlR1-WT protein. (**B**) Chromatographic profiles of ChlR1-WT protein from a HiPrep 16/60 Sephacryl S-300 HR column. (**C**) Chromatographic profiles of standard proteins on a HiPrep 16/60 Sephacryl S-300 HR column. The equation of protein molecular weight is shown in the upper right corner. (**D**) Fourteen fractions were selected from the peak area and analyzed by 10% SDS-PAGE. The gel was stained with Coomassie blue. (**E**) The fractions in D were immunoblotted with an anti-FLAG antibody. (**F**) Total protein before size exclusion chromatography (SEC), and fractions 4 and 5 after SEC, were subjected to helicase assay using 0.5 nM duplex DNA substrate.

Certain helicases may self-assemble to form dimers or higher order oligomers, which can influence their catalytic or biological functions [1,7,48]. The Q motif in FANCJ helicase regulates its dimerization in solution [49], but the oligomeric state of ChlR1 has never been determined. To determine whether the Q motif might affect oligomerization of ChlR1, we analyzed the recombinant ChlR1 proteins by size exclusion chromatography. The purified ChlR1-WT protein (**Fig 7A**) was applied to a Sephacryl S-300 HR column, and a major peak was detected at an elution volume of ~65 mL (**Fig 7B**). Using protein standards, we generated a calibration curve (**Fig 7C)** and determined the molecular weight of this peak is ~141.1 kDa, which is close but greater than ChlR1’s expected mass (101.7 kDa); this difference in mass may be due to post-translational modification. We then selected 14 fractions from the peak region for SDS-PAGE (**Fig 7D**) and Western-blot assays (**Fig 7E**), and both results demonstrated that the absorption peak was contributed by the ChlR1 protein. Moreover, we selected fractions #4 and #5 for helicase activity analysis, and found that this monomeric form of ChlR1 had unwinding activity (**Fig 7F**); however, they exhibited lower activity than the same protein before size exclusion chromatography, which may be attributed to the overnight size exclusion chromatography procedure.

**Fig 8:**
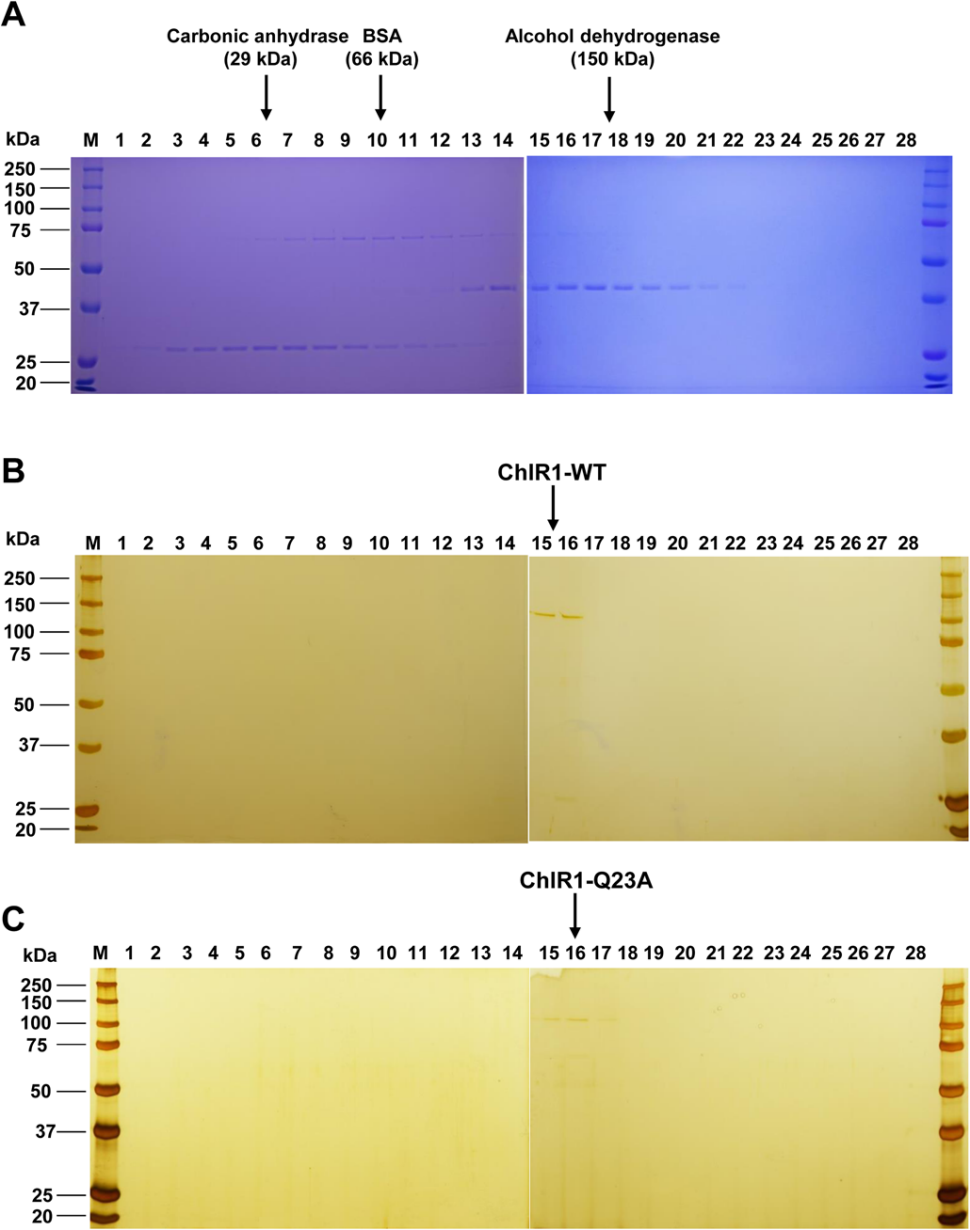
SDS-PAGE analysis of ChlR1 proteins by sucrose gradient fractions. Coomassie blue-stained gels of protein standards (**A**), and silver staining of ChlR1-WT (**B**) and ChlR1-Q23A (**C**) from the sucrose gradient centrifugation. Thirty (30) μL of the sucrose-adjusted fractions (1–28) were loaded per lane. The positions of carbonic anhydrase (29 kDa), BSA (66 kDa), and alcohol dehydrogenase (150 kDa) are indicated at the top. Note that alcohol dehydrogenase is a homotetramer, and is shown in subunits of 37.7 kDa after denaturing.

To evaluate if the presence of ATP affects its apparent oligomerization properties, we incubated ChlR1-WT protein with AMP-PNP, and applied it to a Sephacryl S-300 HR column. A major peak was detected at an elution volume of ~65 mL (data not shown), suggesting ChlR1 protein still exists as a monomer after it is bound by ATP. We also performed sucrose density gradient centrifugation to determine the molecular mass of both ChlR1-WT and -Q23A mutant. SDS-PAGE analysis showed that both ChlR1-WT and Q23A migrated between fractions of BSA (66 kDa) and alcohol dehydrogenase (150 kDa) (**Fig 8**). The molecular mass of the ChlR1-WT was calculated as ~135.4 kDa and ChlR1-Q23A mutant is ~144.2 kDa. Taken together, our results suggest that ChlR1 exists and function as a monomer in solution; this, is different that FANCJ, which can function as both a monomer and dimer [49].

## Discussion

The function of the Q motif has been characterized to include regulation of ATP binding and hydrolysis [9], which further affects substrate binding [25]. However, a recent report suggests that motifs Q and I are required for ATP hydrolysis but not for ATP binding in SWI2/SNF2 protein SMARCAL1 [27]. Thus, the known functions of the Q motif remain inconsistent. We initiated biochemical characterization of ChlR1 and our results suggest that the Q motif in ChlR1 helicase has an essential role in DNA binding but not ATP binding.

Motif Q is also called motif 0, indicating it is a part of the conserved helicase core elements along with seven helicase motifs (I, Ia, II-VI). In fact, experimental evidence shows that the Q motif is the minimal functional unit of the DEAD-box core [50]. In our study, changing the conserved glutamine affected DNA binding in addition to ATP hydrolysis of ChlR1. However, compared with the other seven conserved helicase motifs, the Q motif is somewhat less conserved among SF2 helicases [51]. This motif is absent in the DEAH/RHA and viral DEXH proteins, and these enzymes are not specific for adenosine triphosphates. For example, alanine-scanning mutagenesis has shown that motifs Q, Ia, and Va are not essential for the endonuclease of EcoP15I [22]. Therefore, evidence suggests that the motif Q is more essential for classical helicases, but less or even not essential for other helicase domain-containing proteins.

There are several potential roles for the Q motif. First, Q motif is involved in ATP binding. To be consistent with the initial findings of genetic and biochemical approaches [9,26], several crystal structures show the Gln residue in the Q motif interacts with ATP. In the SF2 UvrB helicase of *Bacillus Caldotenax*, for example, the Gln residue mainly interacts with the adenine base, positioning it for catalysis [14]. Similarly, the Gln residue of *E*. *coli* RecQ helicase [52] and archaeal XPD [53,54] is also located in a RecA domain near the ATP binding cleft. In the crystal structure of *Thermoplasma acidophilum* (*Ta*) XPD solved in the presence of a small fragment of bound DNA, the Q-motif stabilizes the P-loop via hydrogen bonding, which also suggests a role in nucleotide binding and positioning [55]. Secondly, the Q motif is involved in substrate binding. The current work and our previous work on FANCJ [49] suggest that the Q motif is involved in DNA binding. The co-crystal structure of *Archaeoglobus fulgidus* Hel308 with a partial duplex DNA molecule placed the Q motif on the edge of RecA domain 1 near the cleft that is formed with RecA domain 2 [56]. Third, the Q motif might be involved in both ATP and nucleic acid binding. A study of *Saccharomyces cerevisiae* Ded1 helicase demonstrated that the Q motif regulates not only ATP binding and hydrolysis but also the affinity of the protein for RNA substrates [25]. To address whether conformal change by ATP binding will affect the nucleic acid binding that was observed in Ded1 helicase, we incubated ChlR1-Q23A with AMP-PNP, then DNA substrate, and found its DNA binding activity was reduced (**S2 Fig**). Lastly, the Q motif might be involved in protein oligomerization. The oligomerization of human RECQ1 helicase is mediated by both an N-terminal domain (residues 1–103) that bears the conserved Gln residue (amino acid 96) and a C-terminal Winged helix domain [57].

Although helicases share similarities in their three-dimensional folds, particularly the two RecA-like domains [58–60], the human protein and its bacterial counterpart may have subtle differences in structure/function. No ChlR1 ortholog has been found in *E*. *coli*; rather, the highest homology protein in *E coli* is DinG [61]. So far, the structure for neither DinG nor human Rad3/XPD has been solved. The structure of *Thermoplasma acidophilum* XPD, the closest ChlR1 homolog from archaea, in complex with a short DNA fragment has been solved [55]. However, the conserved glutamine (Q8) of Q motif is not directly interacting with DNA (**S4A Fig**). In order to obtain insight into the structural function of Q motif in the ChlR1 helicase, we superimposed ChlR1 sequence on the *Ta*XPD structure (PDB 4A15, **S4B Fig**). The predicted ChlR1 model started from residue Glu202, while the *Ta*XPD structure begins with Arg5. Because full-length ChlR1 has 906 amino acids while *Ta*XPD has only 620 amino acids, about 180 amino acids between motif I and motif Ia are missing in the *Ta*XPD sequence (**S4C Fig**). Thus, the Q23 residue cannot be located in the predicted ChlR1 model and, hence, the predicted ChlR1 model cannot truly illustrate the function of the Q motif in the ChlR1 helicase. Furthermore, secondary structure prediction revealed that the sequence between motif I and motif Ia in ChlR1 is able to form both α-helixes and β-sheets (**S4D Fig**). Therefore, we cannot rule out the possibility that the Q motif in ChlR1 may have a different role; the Q motif of *Ta*XPD does not contact directly the DNA. Taken together, we believe that additional sequence motifs in human ChlR1 than exist in the corresponding homologs from archaea might contribute to its extra structure and function.

Similar to ChlR1, FANCJ (also known as BACH1 or BRIP1) is also a member of the SF2 DEAH-box DNA helicase family [62]. Previous work reports the FANCJ-Q25A mutant is able to bind ATP, as suggested by similar *Km* values compared to the wild-type protein; however, the mutant protein is clearly compromised in its ability to hydrolyze ATP [49]. The reduced ATPase activity of FANCJ-Q25A may be partly attributed to its reduced ability to bind DNA. Our results also demonstrate that the ChlR1-Q23A mutant has poor DNA binding ability, suggesting the role of the Q motif present in FANCJ and ChlR1 (involvment in DNA binding) is conserved. Because ATPase activity is usually DNA dependent, we hypothesized ChlR1-Q23A abolished the ATP hydrolysis activity due to its poor DNA binding ability. In further ATP binding assays, we found that ChlR1-Q23A protein has normal ATP binding ability compared with ChlR1-WT. Thus, similar to the Q motif in FANCJ, the ChlR1 Q motif is also not essential for ATP binding.

DNA helicases appear to be generally oligomeric (usually dimers or hexamers), which provides the helicase with multiple DNA binding sites [1], this process can influence their catalytic or biological functions. Oligomerization is essential for ring-shaped helicases (RepA, Rho) to have function [63]. Even though many non-ring-shaped SF1 and SF2 helicases function as monomers, their activity is greatly enhanced by the formation of dimers or higher oligomers [64–66]. The oligomerization state of ChlR1 is unknown, whereas biochemical studies have demonstrated that the optimal assembly state of FANCJ for catalytic activity is a dimer; the Q motif regulates its dimerization [49]. TaXPD is believed to exist as a monomer in solution [67], whereas eukaryotic XPD is a stable component of the multi-subunit general transcription factor TF IIH complex [68]. So, it is important to examine the ChlR1 oligomerization state as it may influence substrate specificity or catalytic efficiency. We used gel-filtration and sucrose gradient centrifugation, and determined that ChlR1 protein exists as a monomer; further helicase analysis showed this monomeric form has unwinding activity. In fact, another Rad3/XPD family member archaeal XPD function as a monomer in solution to unwind dsDNA substrate, with no known stable interactions with other proteins [69]. Interestingly, the Q motif in FANCJ is involved in protein dimerization; however, ChlR1 functions as a monomer in solution. However, ChlR1 does not form oligomers so the effect of the Q-motif cannot be rationalized.

In conclusion, the conserved glutamine in the Q motif has been shown to be required for ATP binding, though this is not universally the case. We have discovered that the Q motif in ChlR1 is essential for its DNA binding and helicase activity. Because all ChlR1-like helicases possess a Q motif (**S5 Fig**), it will be of interest to determine whether mutations in the Q motif affect protein interactions in other helicases of this family and related SF2 families. Mutational analysis of the Q motif in ChlR1 helicases and related helicases will provide insight into the roles of the Q motif in superfamily 2 DNA helicases, which play vital roles in DNA replication, repair, recombination, and transcription.

## Supporting Information

S1 Fig Alignment of human ChlR1 orthologues across species. The invariant glutamine in the Q motif and helicase motif I were highlighted with yellow.
(PDF)

S2 Fig DNA binding of wild-type ChlR1 proteins as detected by EMSA. The indicated concentrations of ChlR1-WT protein were incubated with 0.5 nM forked duplex DNA under pre-incubation with AMP-PNP (2 nM final concentration for 15 min, left side) or without AMP-PNP (right side) at room temperature for 30 min as described in “Materials and methods”. The DNA-protein complexes were resolved on native 5% polyacrylamide gels.
(PDF)

S3 Fig ChlR1-Q23A fails to unwind short DNA triple helixes. Helicase reactions (20 μl) were performed by incubating the indicated ChlR1-WT (**A**) or ChlR1-Q23A (**B**) concentrations with 0.5 nM 5’ tail flush triplex substrate at 37°C for 20 min under standard helicase assay conditions as described under “Materials and methods”. *Triangle*, heat-denatured DNA substrate control.
(PDF)

S4 Fig The structural model of human ChlR1 protein. (**A**) A side view of the TaXPD–DNA structure (PDB: 4A15). Single stranded DNA is indicated in red. The glutamine (Q8) is highlighted in blue. (**B**) Superimposing ChlR1 (orange) on a TaXPD helicase structure (green). The structure was made with program SWISS-MODE and viewed with Swiss PdbViewer 4.0. (**C**) Sequence alignment of the N terminal region of hFANCJ (top), hChlR1 (middle) and TaXPD (bottom). The grey bars indicate the homologous residues. The alignment was generated with Clustal X. (**D**) The predicated secondary structure for ChlR1 N terminus (1–200 aa). The pink columns represent α-helix, yellow arrows represent β-sheet, and blue bars indicate the confidence of prediction. The position of Q23 in ChlR1 is indicated.
(PDF)

S5 Fig Alignment of human ChlR1-like helicases. The invariant glutamine in the Q motif and helicase motif I were highlighted with yellow.
(PDF)

S1 Table DNA oligomers used in this study. (PDF)
