# Supplementary material for: The Q Motif Is Involved in DNA Binding but Not ATP Binding in ChlR1 Helicase
Source: PLoS One. 2015 Oct 16;10(10):e0140755. doi: 10.1371/journal.pone.0140755 (PMC4608764; doi:10.1371/journal.pone.0140755)
Supplement: S1 Fig — The invariant glutamine in the Q motif and helicase motif I were highlighted with yellow. (PDF) [file pone.0140755.s001.pdf]

Fig. S1

Q **Motif I**

Homo sapiens	1	MANETQKVGAIHFPFPFTPYSIQEDFMAELYRVLEAGK--IGIFESPTGTGKSLSLICGAL	59
Nomascus leucogenys	1	MANETQKVGAIHFPFPFTPYSIQEDFMAELYRVLEAGK--IGIFESPTGTGKSLSLICGAL	59
Pan troglodytes	1	MANETQKVGAIHFPFPFTPYSIQEDFMAELYRVLEAGK--IGIFESPTGTGKSLSLICGAL	59
Ornithorhynchus anatinus	1	MAGPTEEPGRVHFPFPFTPYPIQESFMAELYRVLEAGK--IGIFESPTGTGKSLSLICGAL	59
Equus caballus	1	MANKTPEVGDIHFPFPFTPYSIQKDFMAELYQVLEAGK--IGIFESPTGTGKSLSLICGAL	59
Canis lupus familiaris	1	MSNKTQEIGDIHFPFPFTPYSIQKDFMAELYRVLEAGK--IGIFESPTGTGKSLSVICGAL	59
Bos grunniens mutus	1	MADETQEAGGIHFPFPFTPYAIQKDFMAALYQVLEAGK--IGIFESPTGTGCLICGALSWL	59
Gallus	38	CPQAVSGPGRASFPFPYTPYRIQEQFMAALYAALEAGR--IGIFESPTGTGKSLSLICGAL	97
Rattus norvegicus	1	MAEESQEMGGIHFPFPFPPYPIQKDFMAELYKVLEAGK--IGIFESPTGTGKSLSLICGAL	59
Cricetulus griseus	1	MAEESQEIGGIHFPFPFPPYPIQKDFMAELYKVLEAGK--IGIFESPTGTGKSLSLICGAL	59
Mus musculus	1	MADENQEIGGIHFPFPFPPYPIQKDFMAELYKVLEGGK--IGIFESPTGTGKSLSLICGAL	59
Monodelphis domestica	1	MADQIQEGGIHFPFPYTPYSIQKDFMTELYHVLEAGK--IGIFESPTGTGKSLSLICGAL	58
Xenopus ropicalis	1	MDASIVDPAALTFPFPYEPYPIQEQFMEKLYQALEAGK--VGIFESPTGTGKSLSLICGAL	59
Danio rerio	1	MESKNGRFPFPFQPYPIQESFMEALYTALDQRK--VGIFESPTGTGKSLSLICGAL	54
Tribolium castaneum	1	MEVPNNFEFPFQPYPIQHAFMRNLFEVIENKK--FGIFESPTGTGKSLSILCGAI	53
Harpegnathos saltator	1	MELPQEFPFPFPAYEIQKQFMKELYNCLEGGK--LGLFESPTGTGKSLSLICGAL	53
Crassostrea gigas	21	MEEGEGVQELELLDFPFPFKPYDVQKKFMENLYLCLEKGQ--VGIFESPTGTGKSLSLICGAL	82
Ciona intestinalis	3	QHAQESYRLAAPSKFAFPFEPYSIQVDFMKSLYHAIEDKK--IGIFESPTGTGKSLSLICGSL	63
Aegilops tauschii	1	MPPPPPPRQDFPAFPFAPYPIQSEFMSFLYSALSSGPRALALLESPTGTGKTLSIICSAL	58
Triticum urartu	1	MPPPRQDFPAFPFAPYPIQSEFMSFLYSALSSGPRALALLESPTGTGKTLSIICSAL	55
Jatropha curcas	1	MENEENEPKFPGFPYKPYSIQMDFMKALYRSLDKGG--VSMLESPTGTGKTLSIICSSL	55
Saccharomyces cerevisiae	1	MDKKEYSETFYHPYKPYDIQVQLMETVYRVLSEGK-KIAILESPTGTGKTLSLICATM	55
Schizosaccharomyces pombe	1	MCHSKEVKFKTNFHHPYTPYDIQLEFMRSLYSSISDGK--IGIFESPTGTGKSLSLICASL	57
Pseudozyma Antarctica	13	PGATSDPTLASRDFSFPYPQAYSIQLDLMRQVFSTIEDGK--VGLFESPTGTGKSLSLICAAF	73
Cryptococcus gattii	6	AKSATPSLSTPETFPFPYPKPYDIQLDLMRVVFRAIEDGK--IAIVESPTGTGKSLSLLTSTL	66
