# Supplementary figures and images for: The Q Motif Is Involved in DNA Binding but Not ATP Binding in ChlR1 Helicase
Source: PLoS One. 2015 Oct 16;10(10):e0140755. doi: 10.1371/journal.pone.0140755 (PMC4608764; doi:10.1371/journal.pone.0140755)

**Fig. S2**

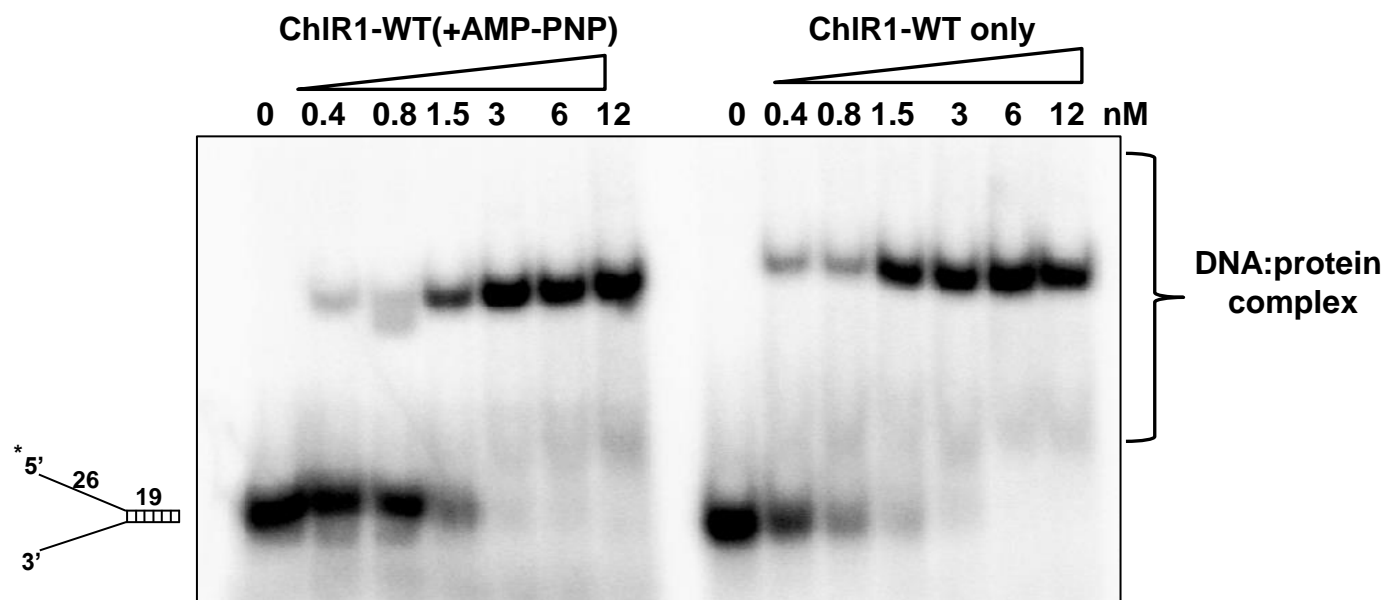

Supplement: S2 Fig — The indicated concentrations of ChlR1-WT protein were incubated with 0.5 nM forked duplex DNA under pre-incubation with AMP-PNP (2 nM final concentration for 15 min, left side) or without AMP-PNP (right side) at room temperature for 30 min as described in “Materials and methods”. The DNA-protein complexes were resolved on native 5% polyacrylamide gels. (PDF) [file pone.0140755.s002.pdf]

**Fig. S3**

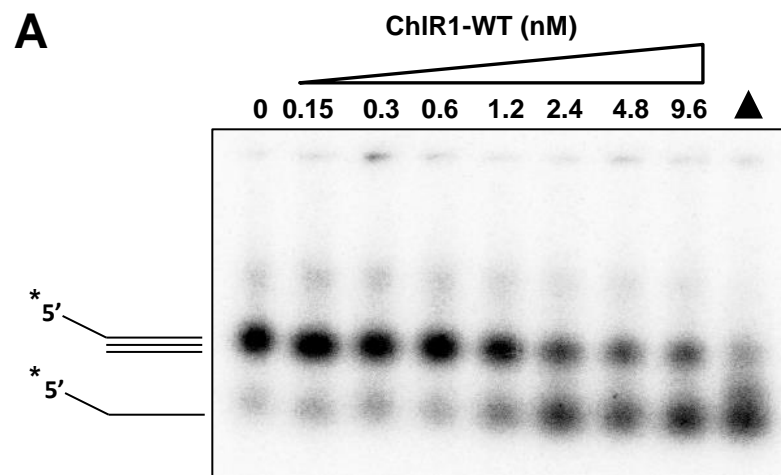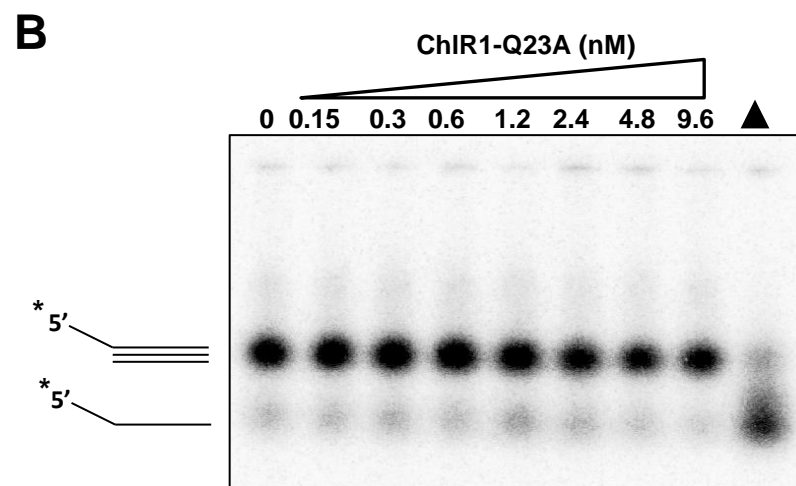

Supplement: S3 Fig — Helicase reactions (20 μl) were performed by incubating the indicated ChlR1-WT (A) or ChlR1-Q23A (B) concentrations with 0.5 nM 5’ tail flush triplex substrate at 37°C for 20 min under standard helicase assay conditions as described under “Materials and methods”. Triangle, heat-denatured DNA substrate control. (PDF) [file pone.0140755.s003.pdf]

**Fig. S4**

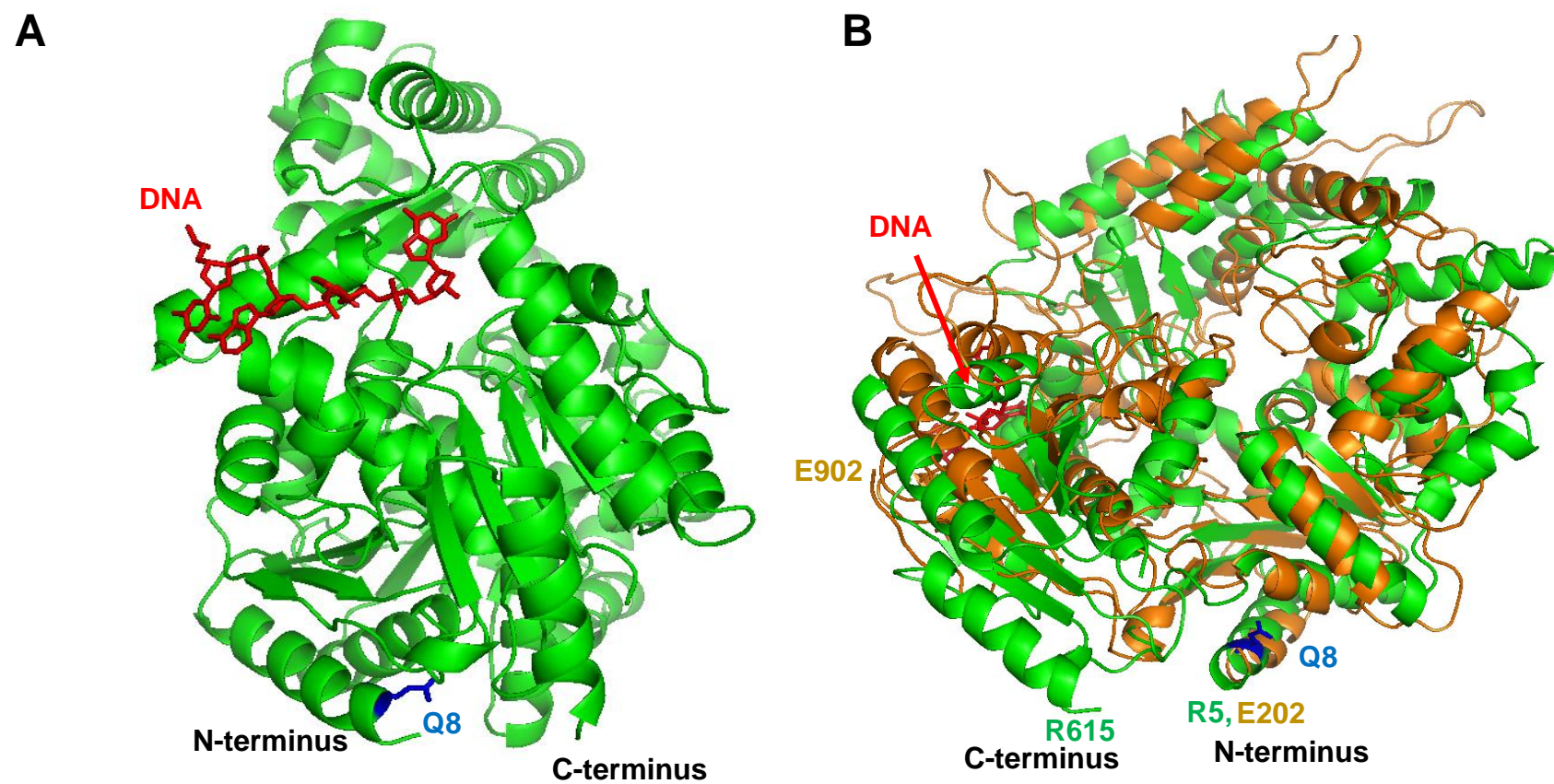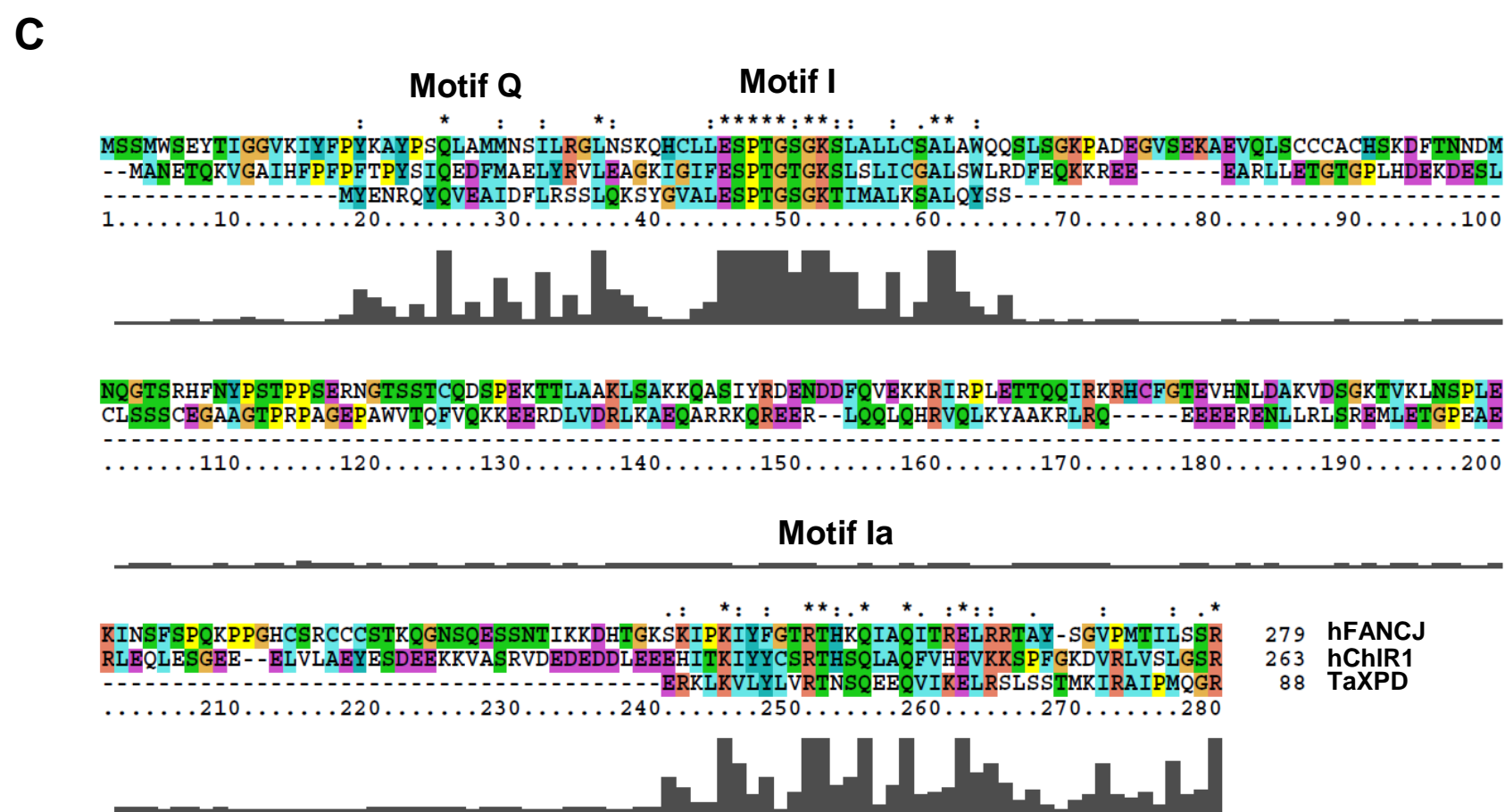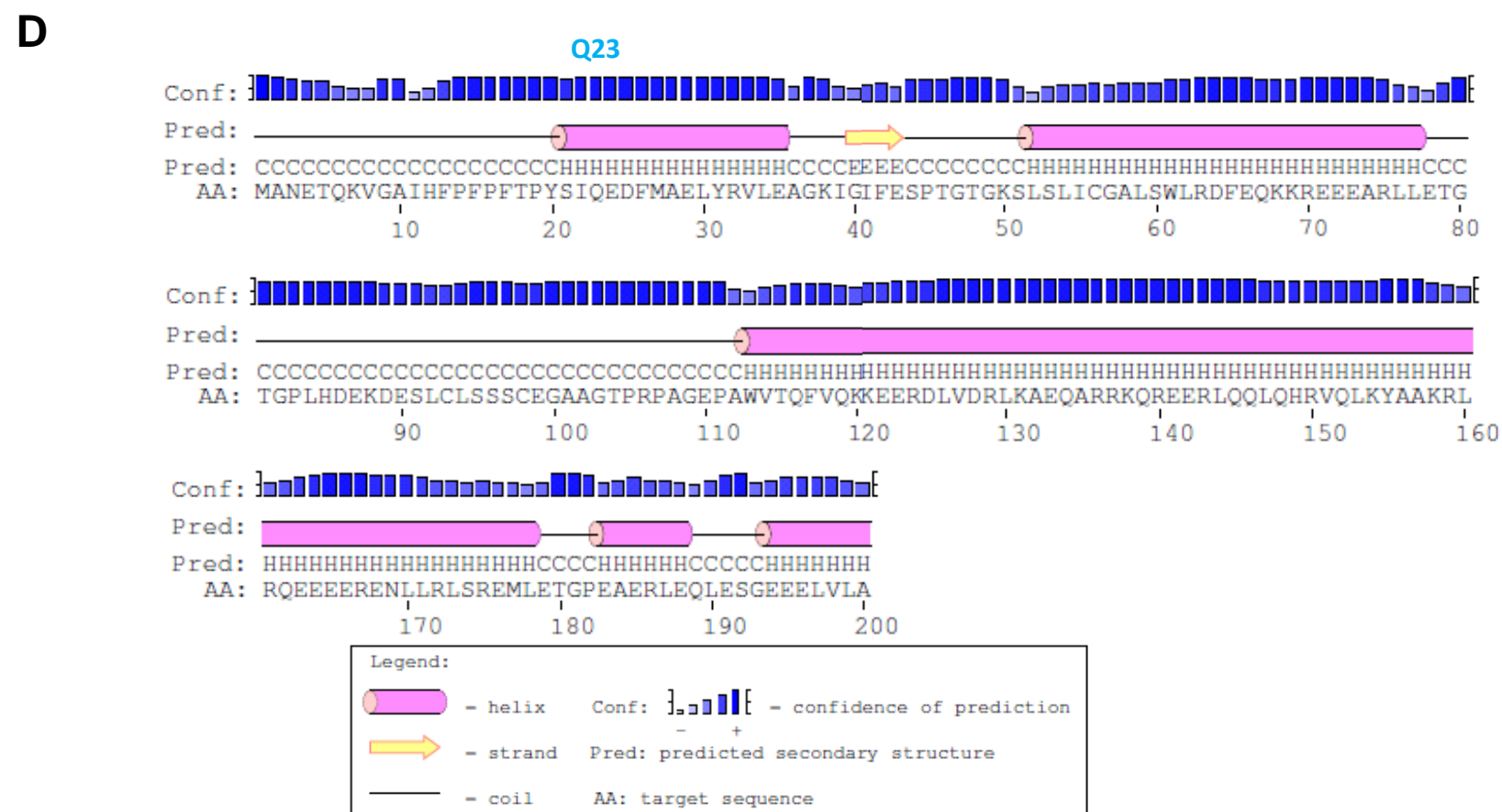

Supplement: S4 Fig — (A) A side view of the TaXPD–DNA structure (PDB: 4A15). Single stranded DNA is indicated in red. The glutamine (Q8) is highlighted in blue. (B) Superimposing ChlR1 (orange) on a TaXPD helicase structure (green). The structure was made with program SWISS-MODE and viewed with Swiss PdbViewer 4.0. (C) Sequence alignment of the N terminal region of hFANCJ (top), hChlR1 (middle) and TaXPD (bottom). The grey bars indicate the homologous residues. The alignment was generated with Clustal X. (D) The predicated secondary structure for ChlR1 N terminus (1–200 aa). The pink columns represent α-helix, yellow arrows represent β-sheet, and blue bars indicate the confidence of prediction. The position of Q23 in ChlR1 is indicated. (PDF) [file pone.0140755.s004.pdf]
