# Supplementary material for: The Q Motif Is Involved in DNA Binding but Not ATP Binding in ChlR1 Helicase
Source: PLoS One. 2015 Oct 16;10(10):e0140755. doi: 10.1371/journal.pone.0140755 (PMC4608764; doi:10.1371/journal.pone.0140755)
Supplement: S5 Fig — The invariant glutamine in the Q motif and helicase motif I were highlighted with yellow. (PDF) [file pone.0140755.s005.pdf]

Fig. S5

```
                       Q                    I
ChlR1  1   MANETQKVGAIHFPFPFTPYSIQEDFMAELYRVLEAGKIGIFESPTGTGKSLSLICGAL  59
RTEL1  1     MPKIVLNGVTVDFPFQPYKCQQEYMTKVLECLQQKVNGILESPTGTGKTLCLLCTTL  57
FANCJ  1 MSSMWSEYTIGGVKIYFPYKAYPSQLAMMNSILRGLNSKQHCLLESPTGSGKSLALLCSAL  61
XPD    1     MKLNVDGLLVYFPYDYIYPEQFSYMRELKRTLDAKGHGVLEMPSGTGKTVSLLALIM  57
```
