# Supplementary material for: The Q Motif Is Involved in DNA Binding but Not ATP Binding in ChlR1 Helicase
Source: PLoS One. 2015 Oct 16;10(10):e0140755. doi: 10.1371/journal.pone.0140755 (PMC4608764; doi:10.1371/journal.pone.0140755)
Supplement: S1 Table — (PDF) [file pone.0140755.s006.pdf]

Table S1 DNA oligomers used in this study

**Oligonucleotide/ DNA substrate**	**Structure and sequence (5’-3’)**	**Used in this study**
ChlR1-Q23A-F	CACACCCTATTCCATCGCGGAAGACTTCATGGCAG	Forward primer for Q23A mutagenesis
ChlR1-Q23A-R	CTGCCATGAAGTCTTCCGCGATGGAATAGGGTGTG	Reverse primer for Q23A mutagenesis
Forked duplex	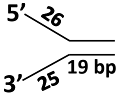 DC26: TTTTTTTTTTTTTTTTTTTTTTCCCAGTAAAACGACGGCCAGTGC<br>T_{STEM}25: GCGGTCCCAAAAGGGTCAGTGCTGGCATTTTGCTGCCGGTCACG	DC26 was either ^{32}P or fluorescence labeled, and used as dsDNA substrate
OX-1 G2’	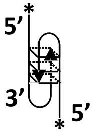 OX-1: ACTGTCGTACTTGATATTTTGGGGTTTTGGGG	A two-stranded antiparallel G4 (G2') substrate
5′ Tail flush triplex	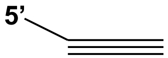 5’Tail TC30: TGACGCTCCGTACGATCTTTTTCTTTCTTT TCTTCTTTTTTCTTT. TC30W: TTTCTTTTTTCTTCTTT TCTTTCTTTTTCT; TC30C: AGAAAAAGAAAGAAAAG AAGAAAAAAGAAA. TC30W and TC30C are used to form duplex DNA, and 5’ tail TC30 as third strand.	A short triplex DNA substrate (flush triplex)
5’Tail plasmid triplex	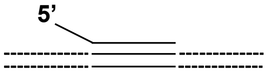 Plasmid pSupF5 is linearized by digestion with *Nde*I, and the third strand is annealed to triplex target site in duplex. 5’ tail TC30 is used as third strand.	A long triplex DNA substrate with 5’tail
